# III-Nitrides Resonant Cavity Photodetector Devices

**DOI:** 10.3390/ma13194428

**Published:** 2020-10-05

**Authors:** Susana Fernández, Fernando B. Naranjo, Miguel Ángel Sánchez-García, Enrique Calleja

**Affiliations:** 1 Energy Department, Centro de Investigaciones Energéticas, Medioambientales y Tecnológicas (CIEMAT), 28040 Madrid, Spain; 2 Grupo de Ingeniería Fotónica (GRIFO), Universidad de Alcalá, 28871 Madrid, Spain; sanchez@isom.upm.es (M.Á.S.-G.); calleja@die.upm.es (E.C.); 3 Instituto de Sistemas Optoelectronicos y Microtecnologia (ISOM) and Electronic Engineering, UPM, 28040 Madrid, Spain

**Keywords:** III-nitrides, vacuum deposition techniques, resonant-cavity optoelectronic devices

## Abstract

III-nitride resonant cavity-enhanced Schottky barrier photodetectors were fabricated on 2 µm thick GaN templates by radio frequency plasma-assisted molecular beam epitaxy. The optical cavity was formed by a bottom distributed Bragg reflector based on 10 periods of Al_0.3_Ga_0.7_N/GaN, an Au-based Schottky contact as top mirror, and an active zone of 40 nm-thick GaN layer. The devices were fabricated with planar geometry. To evaluate the main benefits allowed by the optical cavity, conventional Schottky photodetectors were also processed. The results revealed a planar spectral response for the conventional photodetector, unlike the resonant devices that showed two raised peaks at 330 and 358 nm with responsivities of 0.34 and 0.39 mA/W, respectively. Both values were 80 times higher than the planar response of the conventional device. These results demonstrate the strong effect of the optical cavity to achieve the desired wavelength selectivity and to enhance the optical field thanks to the light resonance into the optical cavity. The research of such a combination of nitride-based Bragg mirror and thin active layer is the kernel of the present paper.

## 1. Introduction

UV-photodetectors based on III−V nitrides are very attractive for a large number of applications, such as flame, pollution and missile monitoring, space-to-space communications and medical and biological effects sensing, since these nitride-based devices are blind to the sun [1,2,3,4,5]. The most important requirements are high visible rejection, high responsivity at the wavelength of interest, low dark current, linear behavior with the incident optical power and low time response. Numerous different architectures of semiconductor-based detectors have been reported in the literature [6,7,8,9]. Each of them has a different working mechanism, cost or characteristics that determine their application field. Among them, the following stand out: Schottky barrier photodiodes, p–i–n photodiodes, metal–semiconductor–metal (MSM) photodetectors or photoconductors for ultraviolet (UV) light detection. Their performance is highly dependent on the semiconductor material used. In this sense, III-nitride semiconductors and/or metallic oxides are the most preferred [1,9,10]. This is because these wide-bandgap materials are chemically and thermally more stable; they have a high breakdown field and a high saturation velocity, and the cut-off wavelength can be chosen by using ternary alloys via modifying the chemical composition. All these exceptional properties lead to the advantages for devices to operate in harsh environments, reduce the downscaling limits and have intrinsic insensitiveness to visible and infrared radiation [1].

As example, AlGaN-based solar blind photodetectors with excellent characteristics have been demonstrated using p-i-n, MSM or Schottky type structures [11,12,13]. However, in these devices, the quantum efficiency is found to be limited by both the thickness and the absorption coefficient of the active layer, in addition to the light reflection on its surface. For this reason, a thicker active layer is required to increase the quantum efficiency value. However, such a thick active region would limit the bandwidth of the device and therefore decrease the speed operation in the device [14]. Therefore, a new design is demanded. In this sense, resonant cavity-enhanced photodetectors (RCE PDs) could be a good choice thanks to the cavity which is based on a very thin absorbing layer sandwiched between two mirrors. Both facts would help to solve the absorption problem, reaching an optical enhancement due to the optical cavity. This enhancement is determined by the quality factor of the cavity. This parameter is determined by the reflectivity of the mirrors that make up the optical cavity, as well as the absorbing layer thickness. Hence, the resonant cavity design can offer several advantages such as wavelength selectivity, the enhancement of the quantum device efficiency and the dependence of the quantum efficiency with the incident light angle [15]. In particular, Schottky RCE PDs are very attractive because of their ease of manufacturing, very low contact resistance and high-speed photodetection [14]. The first Schottky RCE PD developed was reported by Chin et al. [16], based on InGaAs, and with the cavity resonance designed at the wavelength of 0.84 µm. Two years later, Tzeng et al. [17] demonstrated a high-speed operation in RCE PD devices based on GaAs as an active layer. However, it was Kimukin et al. [18] who developed the first AlGaN-based Schottky RCE PDs in the UV range, using an active layer fabricated by metal-organic chemical vapor deposition (MOCVD).

In this work, the great challenge consists in the development of GaN-based Schottky RCE PDs at 360 nm of wavelength detection grown on a GaN template substrate (2 µm-thick GaN grown on sapphire by MOCVD) by plasma-assisted molecular beam epitaxy (PA-MBE). The active layer chosen was a very thin GaN layer because of its moderate absorption coefficient of 3 × 10^−3^ cm^−1^ (measured at room temperature) [19] at the design wavelength. The optical cavity was formed by a bottom distributed Bragg reflector (DBR) based on 10 periods of Al_0.3_Ga_0.7_N/GaN and an Au Shottky contact acting as the top mirror. To demonstrate the wavelength selective detection and the enhancement of the performance due to the cavity, a conventional photodetector was also fabricated for comparison.

## 2. Materials and Methods 

### 2.1. Design of the RCE Photodetector

The main figures of merit to quantify the photodetector performance are photocurrent-to-dark current ratio (PDCR), responsivity (*R*), quantum efficiency (*η*) and response time. PDCR is a measure of the photodetector sensitivity with respect to the dark (or leakage) current, and it is defined as [20]

(1) $$\mathrm{PDCR}\;=\;\frac{I_{photo}-I_{dark}}{I_{dark}},$$

where *I_photo_* is the measured photocurrent and *I_dark_* the dark current. Responsivity is the measurement of photodetector sensitivity defined as the photo-generated current (*I_photo_* − *I_dark_*) per unit of incident optical power *P_opt_* as follows:

(2) $$R(\frac{A}{W})\;=\;\frac{I_{photo}-I_{dark}}{P_{opt}},$$

This parameter is directly proportional to the external quantum efficiency (*η*) that measures the number of photo-generated electron-hole pairs per number of incident photons. The external quantum efficiency is defined as

(3) $$\eta =\;R\frac{hc}{q\lambda^{\prime }}$$

where *c* is the speed of light, *h* is the Plank’s constant, *q* the elementary charge and *λ* the wavelength. Photodetector response time (related to bandwidth) is also quantified in terms of photocurrent 10% to 90% rise time and photocurrent 90% to 10% decay time.

**Figure 1:**
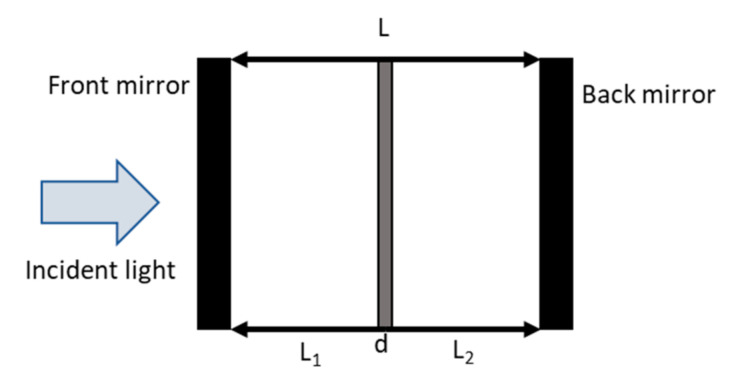
Schematic structure of a cavity of a Schottky resonant photodetector device. *L*_1_ and *L*_2_ are the distances between the active layer and the respective mirrors, and L is the cavity length.

The resonant device has a characteristic whereby the optical field is enhanced because of the light resonance and, because of this, the wavelength selectivity is reached [21]. For this reason, its active layer can be thinner while increasing the quantum efficiency at the resonant wavelengths. The RCE PD design consists of a very thin absorption region of thickness d, embedded between two relatively poorly absorbing layers of thickness L_1_ and L_2_, respectively. The optical cavity is formed by a period of *λ*/4 bottom distributed Bragg reflectors (DBR) with considerably high reflectivity *R*_2_ and a top mirror with lower reflectivity than the back one (*R*_1_ < *R*_2_) that shows a transmittance *T*_1_ high enough to allow the light to travel into the cavity. On the other hand, the reflectivities *R*_1_ and *R*_2_ must provide the optical confinement in the cavity required by this type of device (see Figure 1). In this sense, the trapped light in the optical cavity is absorbed each time that it passes through the absorption region, unlike what happens in a conventional detector, where light is absorbed in a single pass [21,22].

Taking this into account, the quantum efficiency of the RCE PD is a periodic function of λ and reaches the maximum values at resonance wavelengths, defined by [23]

(4) $$\eta_{max}=\left\{\frac{\left(1+R_{2}\right)e^{-\alpha d}}{{\left(1-\sqrt{R_{1}R_{2}}e^{-\alpha d}\right)}^{2}}\right\}\left(1-R_{1}\right)\left(1-e^{-\alpha d}\right)$$

where *R*_1_ and *R*_2_ are the reflectivity of the top and bottom mirrors, respectively, *α* is the absorption coefficient of the active layer and *d*, its thickness. For a thin active layer, αd≪1, and hence *η*_max_ becomes as follows [23]:

(5) $$\eta_{max}=\left\{\frac{\left(1+R_{2}\;(1-\alpha d)\right)}{{\left(1-\sqrt{R_{1}R_{2}}\;\left(1-\alpha d\right)\right)}^{2}}\right\}\left(1-R_{1}\right)\alpha d$$

Expression (5) indicates that a higher quantum efficiency, compared with the conventional design, can be achieved with a thinner absorption region. Among the main critical requirements in the design of the RCE PD, one should consider the high reflectivity that must have the bottom mirror and the moderate thickness of the absorption layer. 

**Figure 2:**
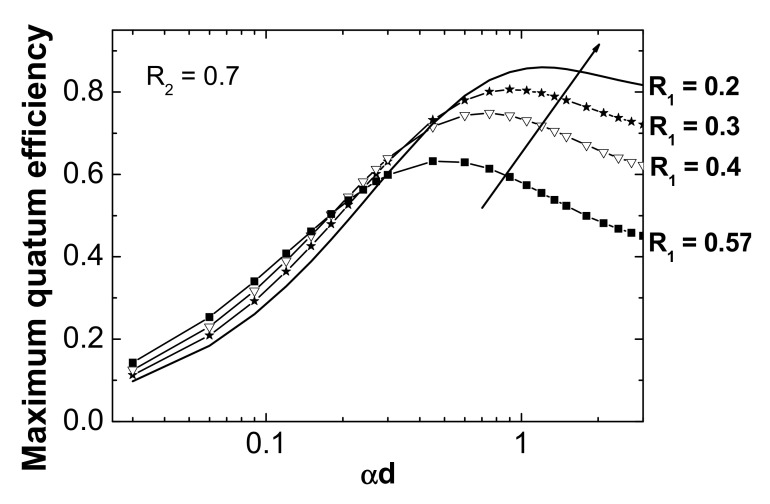
Maximum quantum efficiency versus the normalized absorption coefficient αd at different reflectivity values for the top mirror (*R*_1_) and with *R*_2_ set to 0.7.

In this work, the chosen resonant wavelength was 360 nm; therefore, the active layer was based on a III-nitride binary, GaN, because this material presents a moderate absorption coefficient at the design wavelength [19]. The material used in the cavity to separate the mirrors from the active layer was Al_x_Ga_1−x_N, with a nominal aluminum concentration of 20%. This last choice was made to avoid the absorption at the resonant wavelength and to facilitate the growth of the whole structure by using similar growth temperatures for both materials [24]. The DBR located at the bottom should present high reflectivity without absorption at the design wavelength. In this sense, a DBR based on AlN/Al_x_Ga_1−x_N with low Al content, to present a high index contrast, could be considered as the best theoretical choice. However, from the experimental point of view, the difference between the optimal growth temperatures for both materials is very large, resulting in enormous difficulty to carry it out [25]. This difficulty restricts the back mirror to be constituted by several periods of Al_x_Ga_1−x_N/GaN with nominal Al content close to 30%, having absorption losses at the design wavelength. The choice of Al content is based on the need for this mirror to present high reflectivity values without suffering from structural degradation. For this reason, a moderate number of periods of 10 was chosen and the nominal thicknesses of the III-nitrides layers that made up the DBR were 33.8 nm of GaN and 36.4 nm of Al_0.3_Ga_0.7_N. The next parameter to be determined was the thickness of the GaN active layer. This must be thin enough for light to pass through and reach the back mirror without limiting the response speed of the device. Figure 2 depicts the quantum efficiency versus *αd* for different reflectivity values of *R*_1_ while *R*_2_ is 0.7. As it can be seen, the maximum of the quantum efficiency shifts towards greater values as the product *R*_1_ × *R*_2_ decreases. This indicates that the quantum efficiency peak depends on this product and decreases when increasing the thickness of the absorption layer. The explanation for this trend is based on the fact that a thick absorbent layer would not allow light to pass through the lower mirror, thus eliminating the cavity effect. The maximum quantum efficiency occurs when the following relationship, between the reflectivities of the top and back mirrors, is fulfilled.

(6) $$R_{1}=\left(R_{2}\right)\left(e^{-2\alpha d}\right)$$

In the present work, the theoretical reflectivity of DBR *R*_2_ was estimated to be 0.7 at the design wavelength of 360 nm, and the top mirror was constituted by an Au metal layer with different thicknesses and a theoretical reflectivity ranging from 0.2 to 0.57. Considering these data, the thickness of the GaN absorption layer was chosen to be 40 nm.

**Figure 3:**
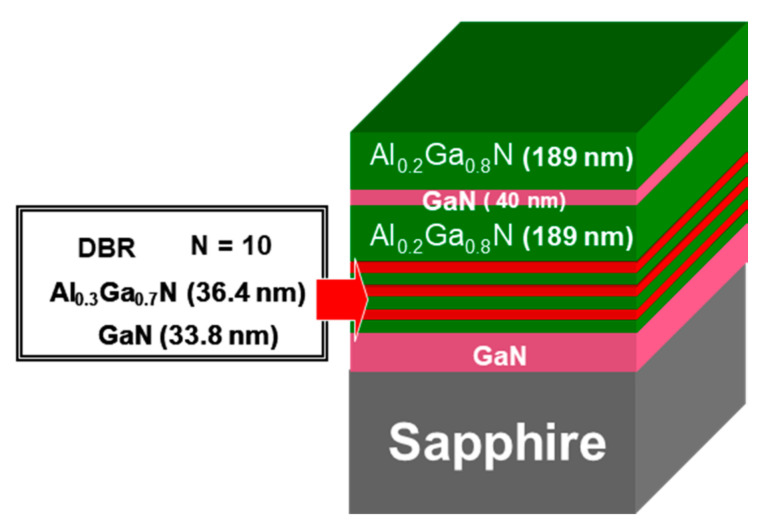
RCE PD design used in this work, where the optical cavity length is 3λ.

The last parameter to be determined was the cavity length L. The chosen optical thickness of the cavity was 3λ [26]. Therefore, the determination of the thickness of the AlGaN layers that embedded the GaN active layer was carried out using the following equation:

(7) $$n_{\mathrm{GaN}}L_{\mathrm{GaN}}+2n_{\mathrm{AlGaN}}L_{\mathrm{AlGaN}}=3\lambda$$

where n_i_, L_i_ (i = GaN, AlGaN) are the refractive index and the thickness of the layers, respectively. Figure 3 depicts the final design of the RCE PD based on the parameters previously calculated.

### 2.2. Fabrication of the III-Nitride Based RCE Photodetectors

**Figure 4:**
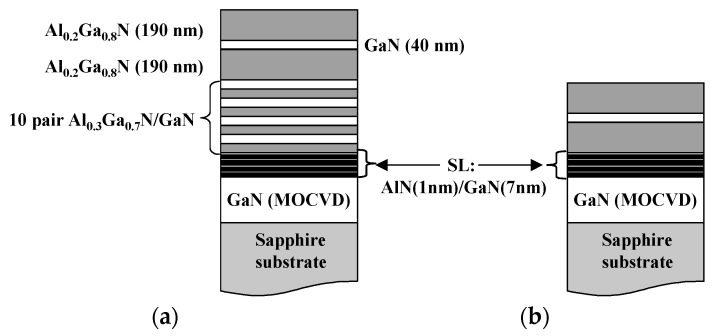
Schematic structure of (**a**) resonant cavity Schottky and (**b**) conventional Schottky photodetectors.

The epitaxial structures were grown on a 2 µm GaN template, using a commercial MECA 2000 MBE system (Vinci technologies S.A., Nanterre, France), where the active nitrogen was supplied from an EPI-UNI Bulb radio frequency plasma source (Veeco Instruments Inc., Sant Paul, MN, USA), and Ga and Al were evaporated using double-filament and cold-lip effusion cells, respectively. In order to compare the performance of the III-nitride-based RCE PDs, a conventional photodetector with the same active zone was also grown. Figure 4 shows the devices fabricated.

In order to prevent the appearance of cracks on the device surface and to avoid its degradation, an AlN (1 nm)/GaN (7 nm) supperlattice (SL) was grown at 660 °C prior to the DBR structure. Subsequently, the bottom mirror of the resonant cavity formed by 10 periods of Al_0.3_Ga_0.7_N (36.4 nm)/GaN (33.8 nm) was centered at *λ* = 360 nm. After this, the GaN layers were deposited at 680 °C, while the AlGaN layers were grown, increasing slightly the growth temperature up to 705 °C. Between the SL and the DBR structure, a growth interruption was carried out. The whole cavity structure was grown at the same growth temperature of 680 °C. A streaky 1 × 1 reflection high energy electron diffraction (RHEED) pattern corresponding to the hexagonal symmetry was observed throughout the GaN growth. This pattern was associated with smooth surfaces; hence, this is indicative that the substrate growth temperature was optimal. During the growth of the AlGaN layers, a slight modulation in the streak intensity evolving to a spotty pattern as growth time increased was observed. The modulation became more pronounced when the Al concentration was raised to 30%. This was a sign that as aluminum concentration increased, the substrate temperature moved farther away from the optimal one. Hence, a slightly rough surface was conferred upon the AlGaN layer. Finally, the streaky 1 × 1 RHEED pattern turned into a 2 × 2 surface reconstruction when cooling down, corresponding to smooth and flat 2D growth, demonstrating that the whole structure was not damaged. More details about the growth can be found in the [25,27].

**Figure 5:**
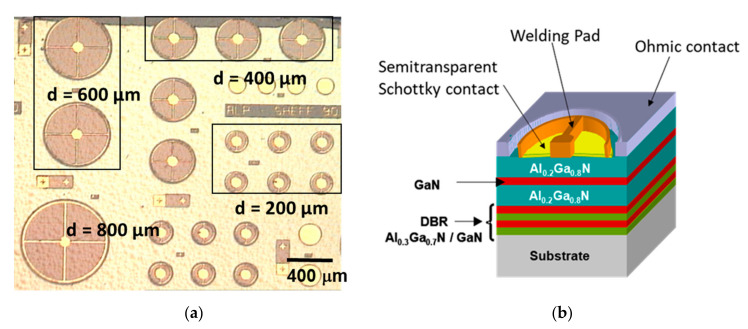
(**a**) Mask of the Schottky photodetectors with planar geometry fabricated with different sizes and (**b**) 3D schematic of the device.

For the fabrication of planar Schottky photodetectors, and prior to the metal deposition, the substrates were immersed in acids (HF:H_2_O 1:10) for 10 sec. Subsequently, an extended Ti (30 nm)/Al (70 nm) contact layer was deposited by electron beam evaporation (e-beam). The metal scheme was annealed at 500 °C for 15 min in nidron atmosphere (95% N_2_ and 5% H_2_). The Schottky contact consisted of Au disks of different thicknesses (75 Å, 100 Å, and 150 Å), and different sizes (from ∅ = 200 µm to ∅ = 800 µm), deposited by joule evaporation, as is pictured in Figure 5. The last step consisted of the deposition of a Ni (30 nm)/Au (100 nm) pad. After this, the sample was placed on a TO-5 package and welding was carried out using Au wires on the semi-transparent contact and Al wires on the extended ohmic one. With this geometry, the detection took place at the top of the surface.

### 2.3. Characterization of the III-Nitride RCE Photodetector

Before the device fabrication, an optical characterization of the DBR was carried out. The reflectivity spectra were measured at room temperature and normal incident with a Perkin-Elmer spectrophotometer (Perkin Elmer Inc., Tres Cantos, Madrid, Spain). These measurements were performed to estimate the quality of growth control and to determine the goodness of the cavity. 

Prior to the welding process, the deposited Schottky contacts were electrically characterized. The quality of the contacts was determined by the current-voltage (I–V) measurements, made with a parameterize in a conventional probe station. The relationship between the applied external voltage *V* and the current I, flowing through the metal-semiconductor junction, can be expressed according to Equation (8) [28]:

(8) $$I\approx exp\left(\frac{qV}{\beta k_{B}T}\right)$$

where *q* is the charge of the electron and *β* is the ideality factor that must be between +1 (ideal diffusion current) and +2 (recombination current). Plotting ln I≈V and adjusting in the straight zone of the I–V characteristic (positive voltages), the ideality factor *β* can be deduced. In addition, under dark conditions, the series resistance and the leakage current can also be obtained. Both parameters are of great importance since they limit the performance of the device. Thus, the series resistance limits the response time of the device and the leakage current is related to the device noise.

Finally, the dependence of the photocurrent with the incident power was measured at room temperature using a non-focused continuous-wave laser line of Ar+ (257 nm). The beam passed through an iris of 1 mm diameter. The intensity of the incident light was obtained from a calibrated sensor located at the position of the resonant photodetector. This measurement was performed under continuous illumination. To obtain the dependence of the detector’s photo response with the irradiance, a variable filter was used that allowed for modifying the optical power of the incident laser beam. On the other hand, the spectral responsivity of the devices was measured using a 150 W xenon arc lamp (Thorlabs Inc., Newton, NJ, USA) and a Jovin-Yvon H-25 monochromator (Jovin-Ybon industrial partner, Richardson, TX, USA). The optical system was calibrated using a Molectron PR200 pyroelectric detector. All devices were characterized at room temperature and at zero bias, connected to a current amplifier with input impedance < 1 kΩ and a gain of about 10^6^ V/A. 

## 3. Results

**Figure 6:**
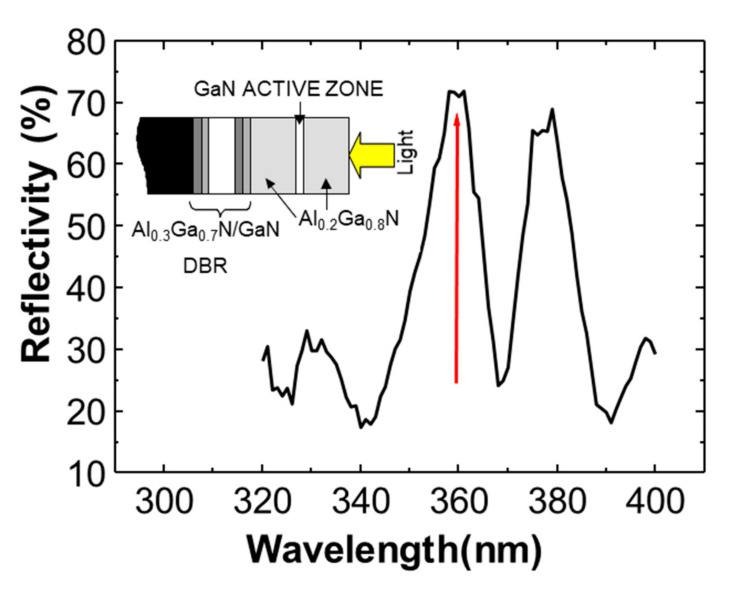
Reflectivity spectrum of the RCE PD measured prior to the device processing.

Figure 6 depicts the reflectivity spectrum of the resonant device prior to its processing. As it can be seen, the spectrum presents a maximum of 72% at the desired wavelength of 360 nm, as was expected. This characterization technique was very useful to confirm that the Bragg mirror was centered at the required wavelength. 

**Figure 7:**
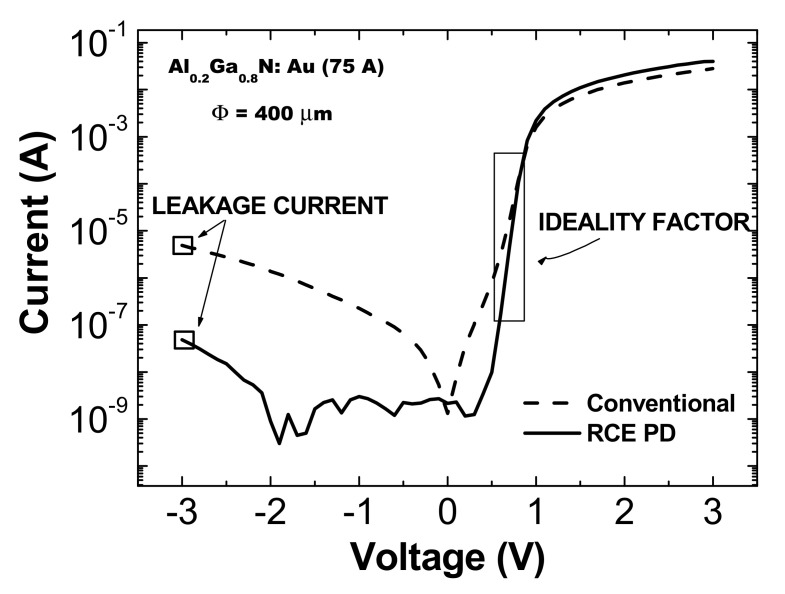
Current-voltage (I–V) characteristics of the conventional (dashed line) and resonant (solid line) photodiodes for 400 µm-diameter devices and a 75 Å-thick Au semitransparent Schottky contact.

Figure 7 shows the I–V characteristics of the Schottky photodiodes of the conventional (dashed line) and 400 µm-size resonant (solid line) devices with a deposited 75 Å-thick Au Schottky contact. It can be noticed that the series resistance of the conventional device was around 100 Ω, while for the resonant device, this parameter was 50 Ω. On the other hand, the leakage current (measured at −3 V) was two orders of magnitude less in the resonant device. 

Table 1 summarizes the ideality factor obtained as function of the device size and the thickness of the Au Schottky contact. The current-voltage (I−V) characteristics present ideality factors ranging from 1 to 4. The expected values should be theoretically between 1 and 2, depending on the dominant effect of the diffusion current or recombination current, respectively [28].

The ideality factors β reached for the resonant photodetectors were lower than for the conventional ones, regardless of the diameter size of the device. In addition, dependence of this factor β on the thickness of the semi-transparent Schottky contact deposited was obtained. Most ideality factors β of the conventional detectors fell outside the theoretical range (>2), suggesting than an extra process made a noteworthy contribution to the conduction. A possible justification for this non-ideality may be the weak Schottky-like nature of the p-contact due to barrier inhomogeneities at the metal–semiconductor interface [29]. 

**Figure 8:**
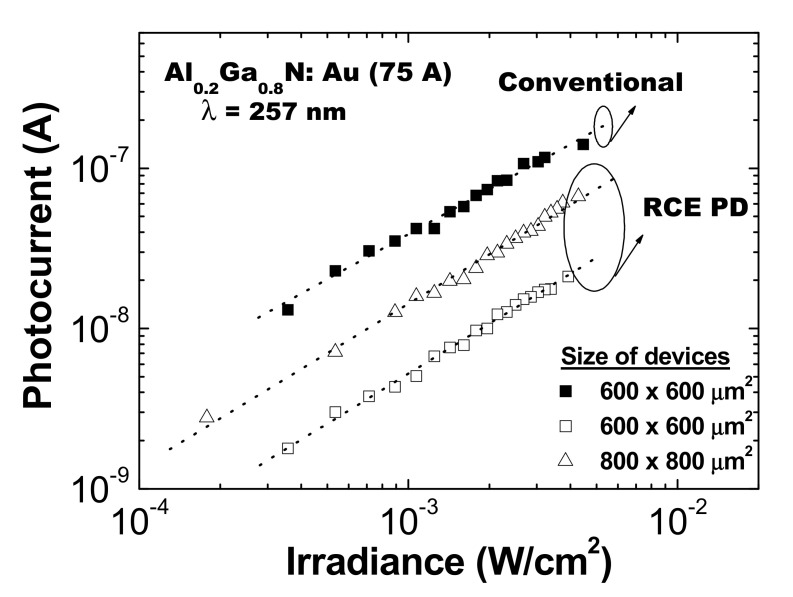
Photocurrent versus illumination power for different conventional and resonant device sizes.

On the other hand, Figure 8 shows the photocurrent as a function of the incident light power. A linear behavior, characteristic of the Schottky barrier photodiodes, was observed regardless of either the type or the size of the devices [30].

**Figure 9:**
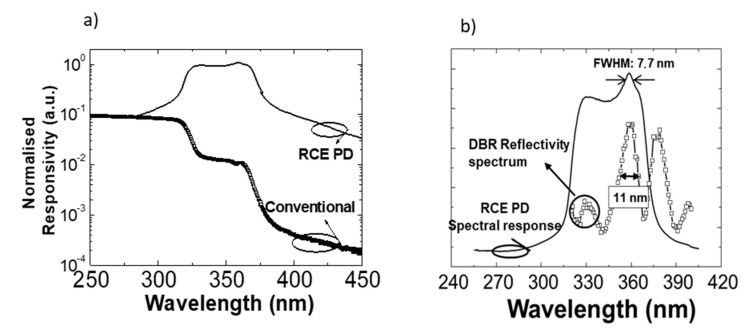
(**a**) Normalized spectral response, from the absorption zone of AlGaN, of conventional and resonant Schottky photodiodes, and (**b**) comparison between the spectral response of the resonant detector and the optical reflectivity spectrum of the device before being processed.

Figure 9a depicts the normalized responsivity of the conventional and resonant Schottky barrier devices. The first one showed a quite flat spectral responsivity for the wavelengths higher than the bandgap of the active zone. An UV/visible contrast of two decades was also obtained for the GaN absorption zone, and four decades for the AlGaN one. In contrast, the responsivity of the RCE PD was not flat, showing two maxima at 330 and 358 nm limited by the full width at half maximum (FWHM) of the main peaks presented in the reflectivity spectrum of the whole device before processing, as is shown in Figure 9b. The main difference between these devices was the shape of the cut-off at the bandgap wavelength, being sharper in the conventional detector, probably due to the great number of defects appeared because of the introduction of the DBR in the resonant structure. The great limitation of using planar geometry was the amount of light reflected in the semitransparent top contact. This fact, together with the growth limitations of the structure, where the DBR was fabricated with absorbent layers at the design wavelength, affected the maximum value of the responsivity, obtaining 0.34 mA/W at 330 nm and 0.39 mA/W at 358 nm in the resonant photodetector, in contrast with a constant value of 0.005 mA/W in the conventional device. 

In addition, the resonant photodiode presented a dark current in the range of 10^−11^ A, an order of magnitude less than that of the conventional one (10^−10^ A). Hence, the photocurrent to dark current ratio of these devices was around 10^3^ for the resonant device, whereas this value was around 10^2^ for the conventional one. This would imply that a 10 times larger photocurrent to dark current contrast ratio could be achieved with the resonant photodiodes, showing their superior performance. These values were close to those reported by several authors in the nitride-based photodetectors field [31].

**Figure 10:**
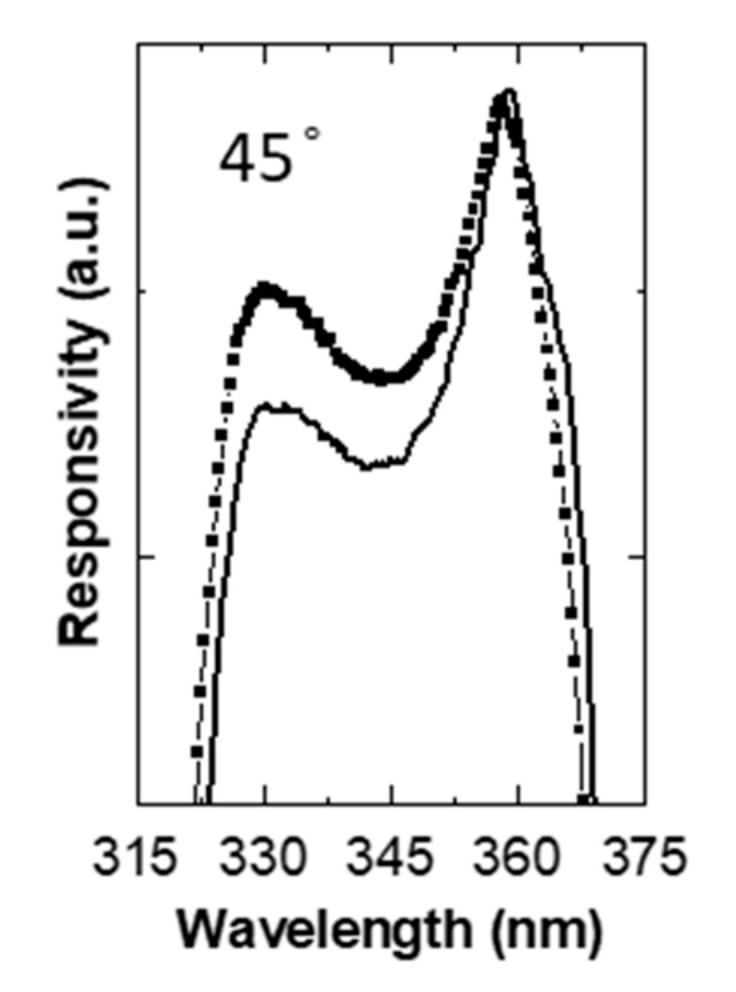
Responsivity spectra for the resonant Schottky photodetector, changing the incident angle of the light.

Finally, to confirm the resonant character of the device, angle spectral responsivity measurements were carried out. For this purpose, it is necessary to demonstrate the displacement of the spectral response as a function of the incidence angle of light [21]. Figure 10 shows the spectral response of the resonant photodiode obtained under illumination at normal incidence (solid line) and at 45° (square symbols). Two effects were observed: (i) a shift in the value of the responsivity maxima to a shorter wavelength and (ii) a change in the intensity ratio between the two peaks. This fact is due to the difference in the length of the optical path when the angle of incidence of the light is changed. 

## 4. Conclusions

We have demonstrated the fabrication of a visible-blind RCE Schottky PD on AlGaN epitaxial layers with lateral geometry grown by MBE. The performance of this device exceeded the conventional device in terms of electric I-V characteristics such as lower ideality factors, lower series resistances, lower leakage and dark currents, independently of the device diameter and the thickness of the Au–Schottky contact. Regarding the optical performance, two maxima at 330 and 358 nm with responsivity values of 0.34 and 0.39 mA/W, respectively, were obtained in comparison with a flat response for the conventional device, whose responsivity value was 70 times lower. The resonant character was also demonstrated.

## Figures and Tables

**Table 1:** Ideality factor β achieved for the fabricated devices.

Type of Device	Device Diameter (µm)	β Au (75 Å)	β Au (100 Å)	β Au (175 Å)
**Conventional**	200	1.5	1.3	2.1
	400	2.2	3.9	1.7
	600	1.9	4.4	2.7
**Resonant**	200	1.4	1.53	1.4
	400	1.2	3.2	1.36
	600	1.2	2.6	2.1
